# Forecasting spatial, socioeconomic and demographic variation in COVID-19 health care demand in England and Wales

**DOI:** 10.1186/s12916-020-01646-2

**Published:** 2020-06-29

**Authors:** Mark D. Verhagen, David M. Brazel, Jennifer Beam Dowd, Ilya Kashnitsky, Melinda C. Mills

**Affiliations:** 1 Leverhulme Centre for Demographic Science, University of Oxford & Nuffield College, Oxford, UK; 2 Interdisciplinary Centre on Population Dynamics, University of Southern Denmark, Odense, Denmark; 3 National Research University Higher School of Economics, Oxford, Russia

**Keywords:** COVID-19, England, Wales, Hospital capacity, NHS, Local, Regional, Ethnicity, Age, Deprivation, Population density

## Abstract

**Background:**

COVID-19 poses one of the most profound public health crises for a hundred years. As of mid-May 2020, across the world, almost 300,000 deaths and over 4 million confirmed cases were registered. Reaching over 30,000 deaths by early May, the UK had the highest number of recorded deaths in Europe, second in the world only to the USA. Hospitalization and death from COVID-19 have been linked to demographic and socioeconomic variation. Since this varies strongly by location, there is an urgent need to analyse the mismatch between health care demand and supply at the local level. As lockdown measures ease, reinfection may vary by area, necessitating a real-time tool for local and regional authorities to anticipate demand.

**Methods:**

Combining census estimates and hospital capacity data from ONS and NHS at the Administrative Region, Ceremonial County (CC), Clinical Commissioning Group (CCG) and Lower Layer Super Output Area (LSOA) level from England and Wales, we calculate the number of individuals at risk of COVID-19 hospitalization. Combining multiple sources, we produce geospatial risk maps on an online dashboard that dynamically illustrate how the pre-crisis health system capacity matches local variations in hospitalization risk related to age, social deprivation, population density and ethnicity, also adjusting for the overall infection rate and hospital capacity.

**Results:**

By providing fine-grained estimates of expected hospitalization, we identify areas that face higher disproportionate health care burdens due to COVID-19, with respect to pre-crisis levels of hospital bed capacity. Including additional risks beyond age-composition of the area such as social deprivation, race/ethnic composition and population density offers a further nuanced identification of areas with disproportionate health care demands.

**Conclusions:**

Areas face disproportionate risks for COVID-19 hospitalization pressures due to their socioeconomic differences and the demographic composition of their populations. Our flexible online dashboard allows policy-makers and health officials to monitor and evaluate potential health care demand at a granular level as the infection rate and hospital capacity changes throughout the course of this pandemic. This agile knowledge is invaluable to tackle the enormous logistical challenges to re-allocate resources and target susceptible areas for aggressive testing and tracing to mitigate transmission.

## Background

COVID-19 is one of the most serious pandemics of the past 100 years, with its rapid global spread overwhelming hospitals and local communities across the world. As of early May 2020, almost 300,000 deaths and over 4 million confirmed cases were registered across 187 countries or regions [1]. Thus far, considerable attention has been focused on understanding basic crude fatality rates (CFRs) and reproductive rates (R_0_) of the infection and their variation by age, sex and underlying medical conditions [2, 3]. Given the high demand for hospitalization and critical care for COVID-19 patients, extensive lockdown measures were enacted in most countries to avoid excessive health care demand, especially hospitalizations. With one-third of patients admitted to the hospital in the UK eventually dying from the virus, unprecedented pressures continue to be placed on health care systems [4]. Hospitalization and death from COVID-19 have been further linked to key socioeconomic and demographic factors, including age, ethnicity, socioeconomic deprivation and population density. Since these factors vary strongly by local and regional area, there is an urgent need to analyse the mismatch between health care demand and supply at the local, fine-grained scale. Infection rates may also vary by location and over time, necessitating a real-time tool for local and regional authorities to flexibly anticipate demand by multiple factors.

With over 30,000 deaths by early May 2020, the UK reached the highest number of recorded deaths in Europe, second in the world only to the USA. After initially discussing a herd immunity strategy, the UK called for social distancing measures starting March 23rd to protect individuals and the health care system from being overwhelmed. At the time of the outbreak, the UK had only 2.5 hospital beds per 1000 population normally available, considerably less than countries such as Italy (3.2), Germany (8.0) or South Korea (12.3) [5]. As part of the government’s response, the allocation and management of health care supply were centralized and seven Nightingale hospitals were erected in urban centres in England (e.g., London, Birmingham) and across the UK (e.g., Cardiff, Glasgow) to address local excess demand for hospital beds relative to normal capacity, further illustrating the key logistical challenges posed by COVID-19 [6].

Analyses of 16,749 patients with severe COVID-19 in 166 UK hospitals from February 6 to April 18, 2020, revealed that patients were older, disproportionally male and more likely to have common comorbidities (cardiac disease, diabetes, asthma) [4]. Although comorbidities and age have been widely acknowledged [3], socio-economic deprivation, living in dense conditions that influence the ability to social distance and links with ethnicity emerged as other core factors underlying risk of COVID-19 hospitalization and death [7, 8]. Crucially, all of these core predictors of COVID-19 hospitalization vary widely across local and regional areas. An added uncertainty is the inability to predict the ultimate infection rate in the population, which even experts continue to adjust to anywhere between 20 to 70% of adults [9]. As lockdown measures start to relax and until a vaccine becomes available, there is an urgent need to anticipate hospital bed demand in new ways and in particular from a geographic perspective. Understanding spatial variations will be key to effective strategic allocation of limited health care resources [10] as well as crucial in informing effective disease monitoring and prevention [11]. The aim of this study is to offer flexible estimates at more fine-grained local and regional levels that take into account multiple socioeconomic and demographic sources of variation in COVID-19-related health care demand and permit a flexible real-time adjustment of the assumed infection rate as local hotspots of infection may arise.

## Methods

### Data

We focus on England and Wales given the steep increase in cases and deaths [1], comparatively late closure of schools, public places and social distancing policies relative to other European countries and lower level of testing and effective contact tracing. For local population counts by age across England and Wales, we used the mid-2018 census estimates at the LSOA level, as estimated by the ONS [12]. Hospital bed capacity for England was taken from NHS’s SDCS data collection for both the general hospitalization bed capacity (collected December 2019) as well as the acute care bed capacity (collected January 2020) [13]. Hospital bed capacity was available per NHS Trust which could be aggregated to both the CCG and CC levels. We geo-coded the postal codes of each NHS Trust into latitude and longitude coordinates to match NHS Trusts to CCG as well. Hospital capacity and locations for Wales were obtained from Statistics for Wales and the NHS Wales Informatics Service [14]. Indices on social deprivation for 2019 were obtained from the Ministry of Housing, Communities & Local Government at the LSOA level for England [15] and from the Welsh government for Wales [16]. The proportion of ethnic minorities per LSOA was obtained from the 2011 census for England and Wales as collected by the ONS [17].

Shapefiles for England and Wales were obtained from the ONS’s Open Geography Portal for the LSOA, CCG and Administrative Region level [18]. We obtained shapefiles for the CC level from Ordnance Survey Digital Data [6]. Lookups between the LSOA and regional level were taken from ONS. Lookups between the LSOA and CC level were obtained by overlaying the shapefiles using the sf package in R. Shapefiles for Welsh Local Health Boards were obtained from the Lle Geo-Portal [18].

### Methods

To inform spatial differences in COVID-19 pressures on the health care system, we study both the capacity and expected demand at a granular level for both general hospitalization, as well as critical care hospitalization. Static maps are shown in this study, with an online companion dashboard available (https://covid19.demographicscience.ox.ac.uk/demrisk), that allows users and policy-makers to examine different geographic levels (region, CC, CCG, LSOA) and adapt estimates by the overall infection rate and hospital capacity relative to normal circumstances. We also offer the possibility to customize estimates with different age-specific infection and hospitalization rates, and bivariate regional maps to examine risks by social deprivation, ethnicity and population density.

As described above, hospital capacity is directly available at various levels of aggregation. To calculate expected hospitalizations per region by age, we aggregated census data at the LSOA level into 5-year age-intervals starting at 0 up until 89. We binned individuals 90 years and older into a single category of 90+ years. For each LSOA, we calculated the number of individuals in every age category and multiplied this number with a fixed, illustrative infection rate of 10%. By using a fixed infection rate across the population, we implicitly assume an equal risk of infection across age and geography. While this assumption is not likely to hold in practice, we are assuming low overall population infection rates relative to other estimates [19] thus illustrating the strong spatial variation in risk across the country even at low levels of infection. The overall infection rate and hospital capacity can be adjusted using the online tool. Our aim is to highlight those areas that may be at a particularly high risk for excess demand relative to others given the same infection spread, even at relatively low levels of infection prevalence. Therefore, modelling a uniform infection spread is logical to illustrate the spatial variations in risk due to local demographic differences. We therefore do not aim to model the possible path of the pandemic but rather to show the strong spatial variation in both hospital capacity as well as expected hospitalization conditional on similar levels of infection spread, with online options offering additional flexibility in estimates.

After calculating the number of infections per LSOA based on our illustrative 10% infection rate, we used estimated COVID-19 hospitalization by age group to calculate the expected number of individuals in need of hospital care, as well as those in need of critical care (with an ability to adjust assumptions online) [19]. By aggregating these estimates up to the Administrative Region, CC, CCG and LSOA level, we estimated the total demand for hospital beds. Total hospital bed capacity was then taken directly from the data described above and we calculated net demand for hospital beds by subtracting estimated demand from actual hospital capacity. We used general hospital and acute bed capacity to calculate relative capacity for general hospitalization and used the acute bed capacity in England and the intensive care capacity in Wales to calculate relative capacity for critical care hospitalization. As mentioned before, these estimates reflect pre-crisis levels of capacity and should be evaluated as the baseline capacity available in a region prior to any additional supply allocations.

Finally, we include a number of bivariate maps where we visualize the age-based general hospitalization risk, as described above, in conjunction with a number of key socioeconomic and demographic factors associated with hospitalization and mortality rates. These include social deprivation, population density and the proportion of ethnic minorities, specifically Blacks and Asians, since recent evidence has shown socially deprived areas and ethnic groups to be particularly hard hit by the crisis [8].

## Results

**Fig. 1:**
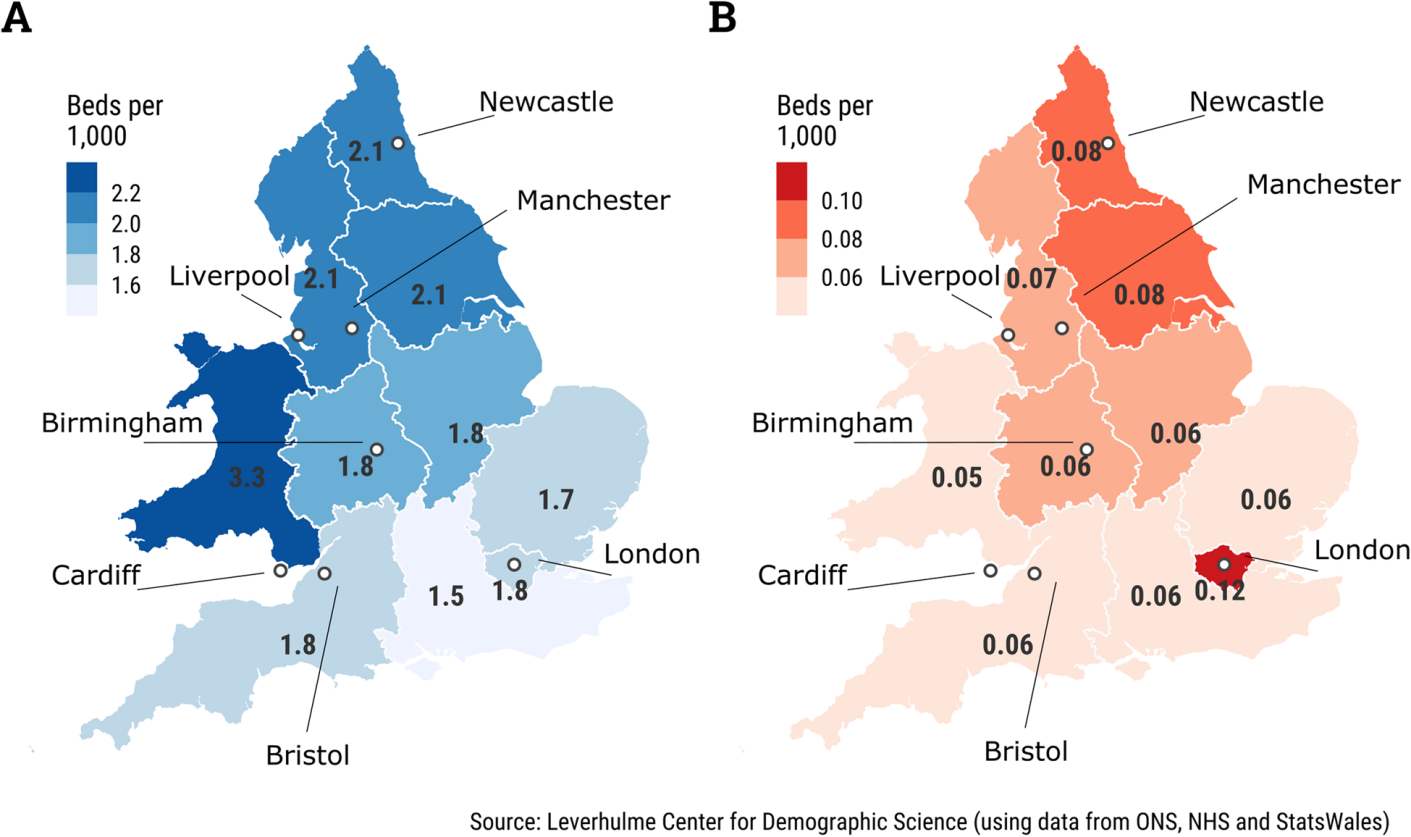
Regional baseline hospital bed capacity (per 1,000) for general care (**a**) and critical care (**b**) in case of a 10% overall infection. England & Wales

**Fig. 2:**
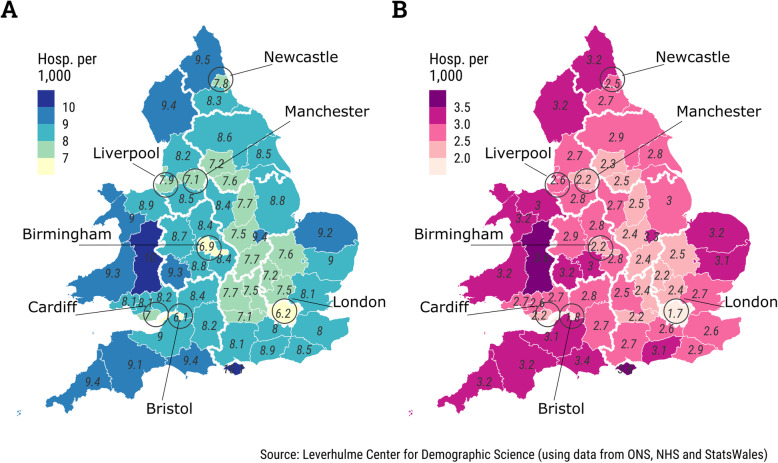
County expected hospitalization (per 1,000) for general care (**a**) and critical care (**b**) in case of a 10% overall infection. England & Wales

We see that the aggregated national average of 2.5 hospital beds in England and 3.3 hospital beds in Wales per 1000 is unequally distributed at both the Administrative Region (Fig. 1), CC (Additional file 1: Fig. S1) and CCG level (Additional file 2: Fig. S2). We consider these rates as the pre-crisis ‘baseline’ available capacity on which the government’s current efforts to expand hospital capacity builds. Hospital capacity can be adapted to reflect 0 to 5 times the capacity relative to this pre-crisis baseline using the online dashboard. Hospitalization, critical care and fatality rates for COVID-19 are all strongly associated with age and underlying comorbidities [2], and age-specific hospitalization rate estimates from recent pandemic modelling of the UK show that hospitalization for those under 40 is expected to be below 3.5% conditional on infection, but rise sharply for older ages, reaching 27.3% for those 80+ [19]. Applying these estimated hospitalization rates across England and Wales, we show that regional demographic variation will lead to stark spatial variation in expected hospitalization rates at the CC (Fig. 2) and CCG (Additional file 3: Fig. S3) level. For example, given our assumption of similar infection rates across regions, we observe very high pressures in rural areas in Wales, as well as the counties of Northumberland and Suffolk in the North-East of England, a disproportionate pressure which is even more serious for critical care beds. Assumptions using alternative infection rates and hospitalization rates can be evaluated using the online dashboard.

**Fig. 3:**
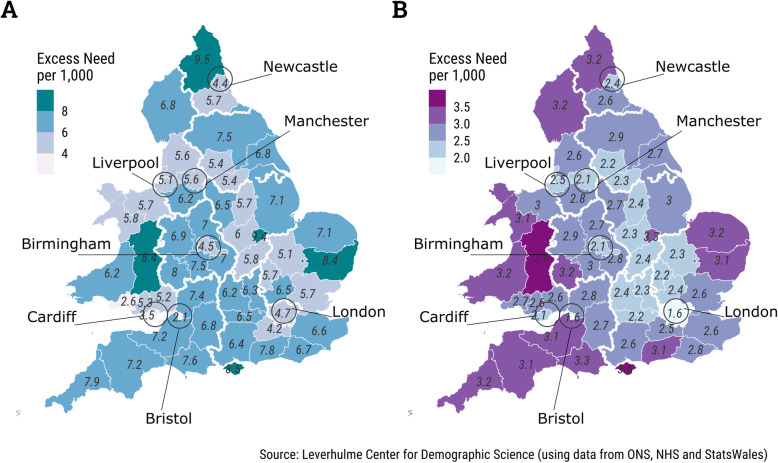
County excess need for hospital beds relative to baseline capacity (per 1,000) for general care (**a**) and critical care (**b**) in case of a 10% overall infection. England & Wales

We also estimate specific pressure points where COVID-19 demand is likely to outstrip the baseline local supply (Fig. 3 and Additional file 4: Fig. S4). This again includes rural areas in Wales as well as the North-East and South-West of England where high expected hospitalization rates combine with relatively low bed capacity. Importantly, these areas are often more isolated and further away from alternative hospital services, meaning excess supply in the direct vicinity will be limited. An example is Cornwall—which lies on England’s South-West coast—where expected hospitalization is not only high, but there are also few counties in the vicinity with relatively low expected hospitalization rates. To illustrate this point further, we geo-coded and visualized all hospitals in Wales together with their general and critical care bed capacity as well as the expected hospitalization rates at the fine-grained LSOA level (Additional file 5: Fig. S5). Hospital capacity is the highest in Cardiff and along the coast, which is logical since they also have the highest levels of population density. Yet it reveals a demographically vulnerable middle rural region of Wales, consisting of an ageing population that are simultaneously far away from hospitals and, in particular, from critical care provisions. Conversely, there is a markedly higher ratio of critical care beds to expected demand in London. This is partly due to its young population structure who are less likely to need critical care, as well as a relatively high supply of critical care beds. It is crucial to note here that some of the more rural areas like Powys in Wales and the Isle of Wight in England have arrangements with neighbouring CCG’s for bed capacity, although they remain relatively far away from the physical care centres. Building on these types of logistical agreements was fundamental in addressing the excessive pressure on the health care system in these regions as the pandemic spread. These agreements differ between regions and are not transparently documented (to our knowledge).

**Fig. 4:**
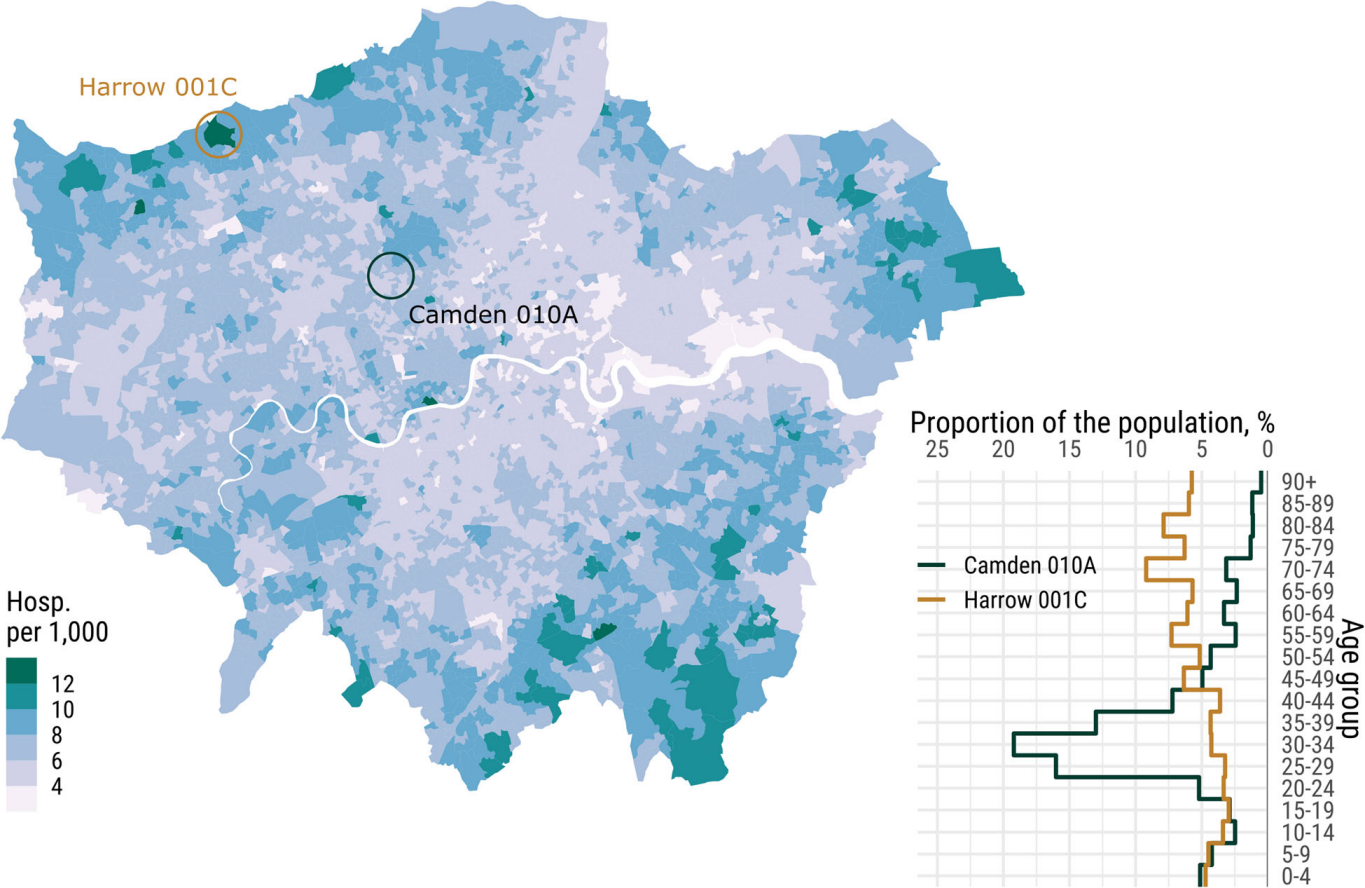
LSOA local differences in expected general care hospitalization (per 1,000) in case of a 10% overall infection. London

Our insights can be used on the Administrative Region, CC and CCG level—as presented above—but can also be expanded to inform decision-making on a more granular local level (LSOA) as well. To exemplify this point, Fig. 4 shows the estimated expected hospitalization rates for London, the current epicentre of the epidemic, on the LSOA level. Even within one city, we still see clear disparities in the estimated rates of hospitalization due to differences in local demographics (Fig. 4). Harrow, for instance, has a much older population than for example Camden, which has comparatively younger inhabitants. Our estimates show that hospital demand from Harrow will be much more severe for a similar infection rate. Our prediction was in fact already realized on March 19, when Northwick Park Hospital in Harrow was the first hospital in London to declare a ‘critical incident’ after running out of intensive care beds [20]. Harrow also had the second highest proportion of deaths over the March to mid-April period for all LSOAs [8]. Whereas urban areas can more easily shift patients to other hospitals, this will be more difficult in rural, sparsely populated areas and areas with ageing populations that may also be experiencing an influx of individuals with second holiday homes.

**Fig. 5:**
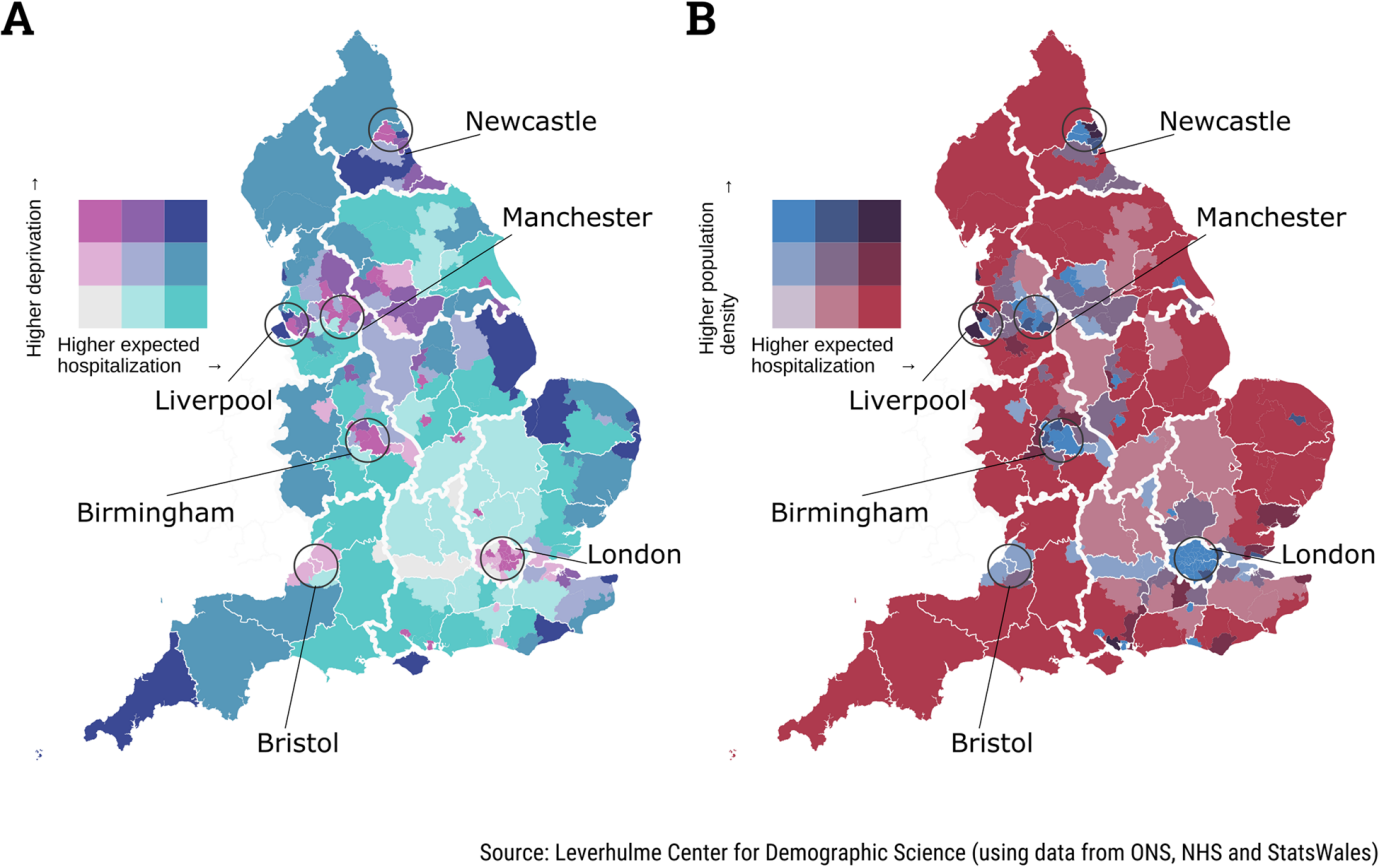
CCG expected age-based hospitalization risk in combination with social deprivation (**a**) and population density (**b**). England

**Fig. 6:**
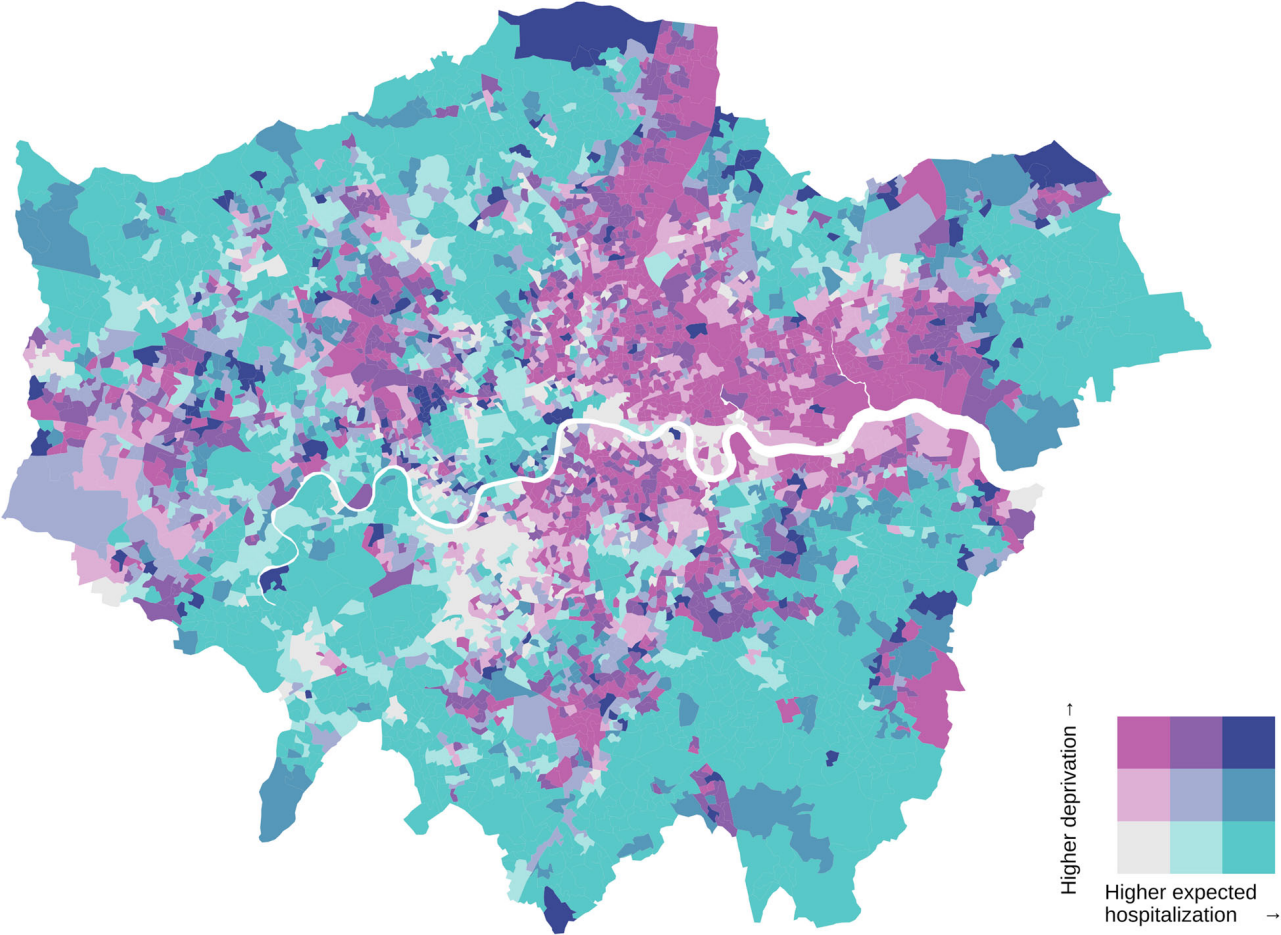
LSOA local differences in age-based hospitalization risk combined with social deprivation. London

**Fig. 7:**
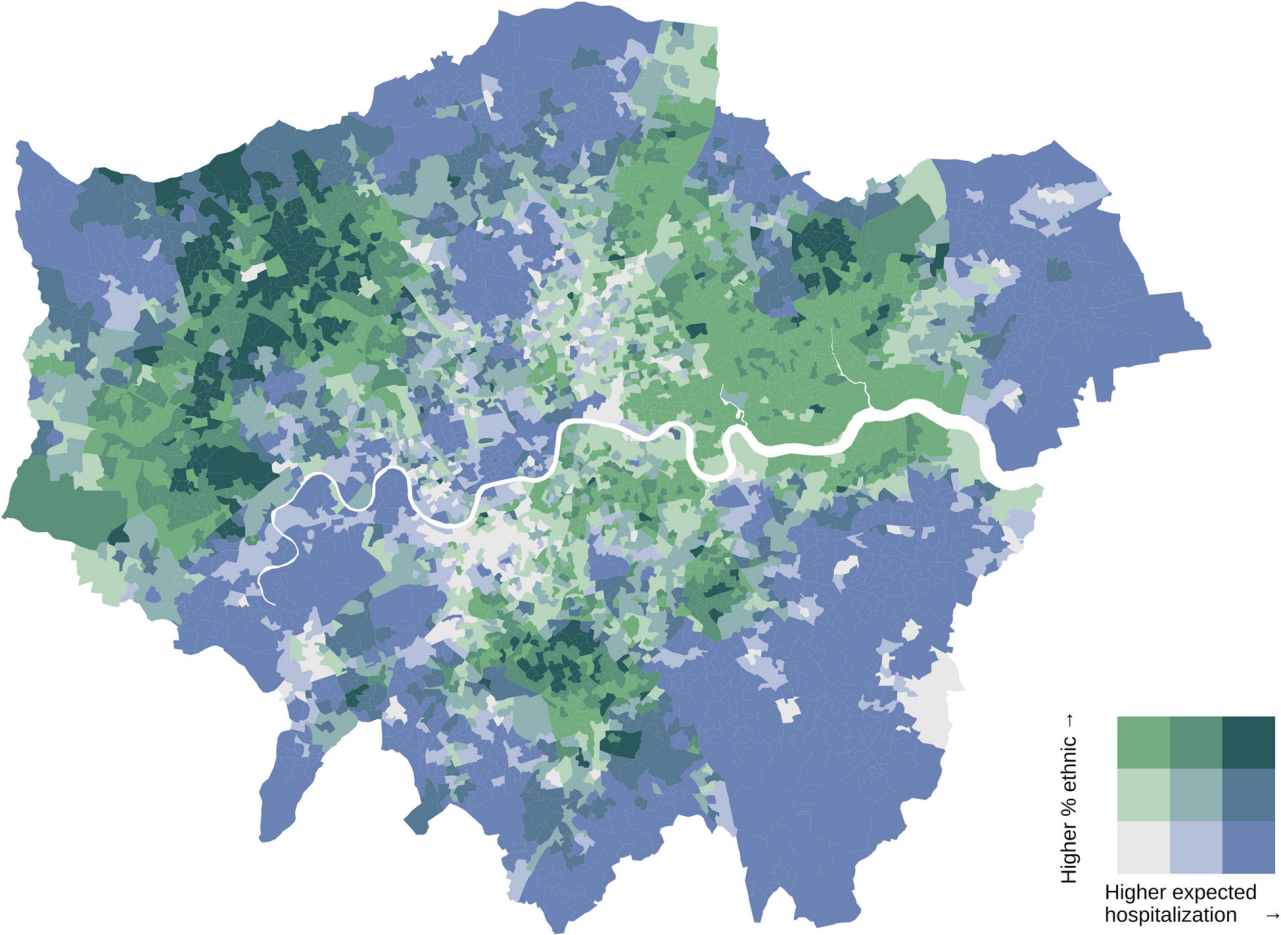
LSOA local differences in age-based hospitalization risk combined with ethnic risk groups. London

**Fig. 8:**
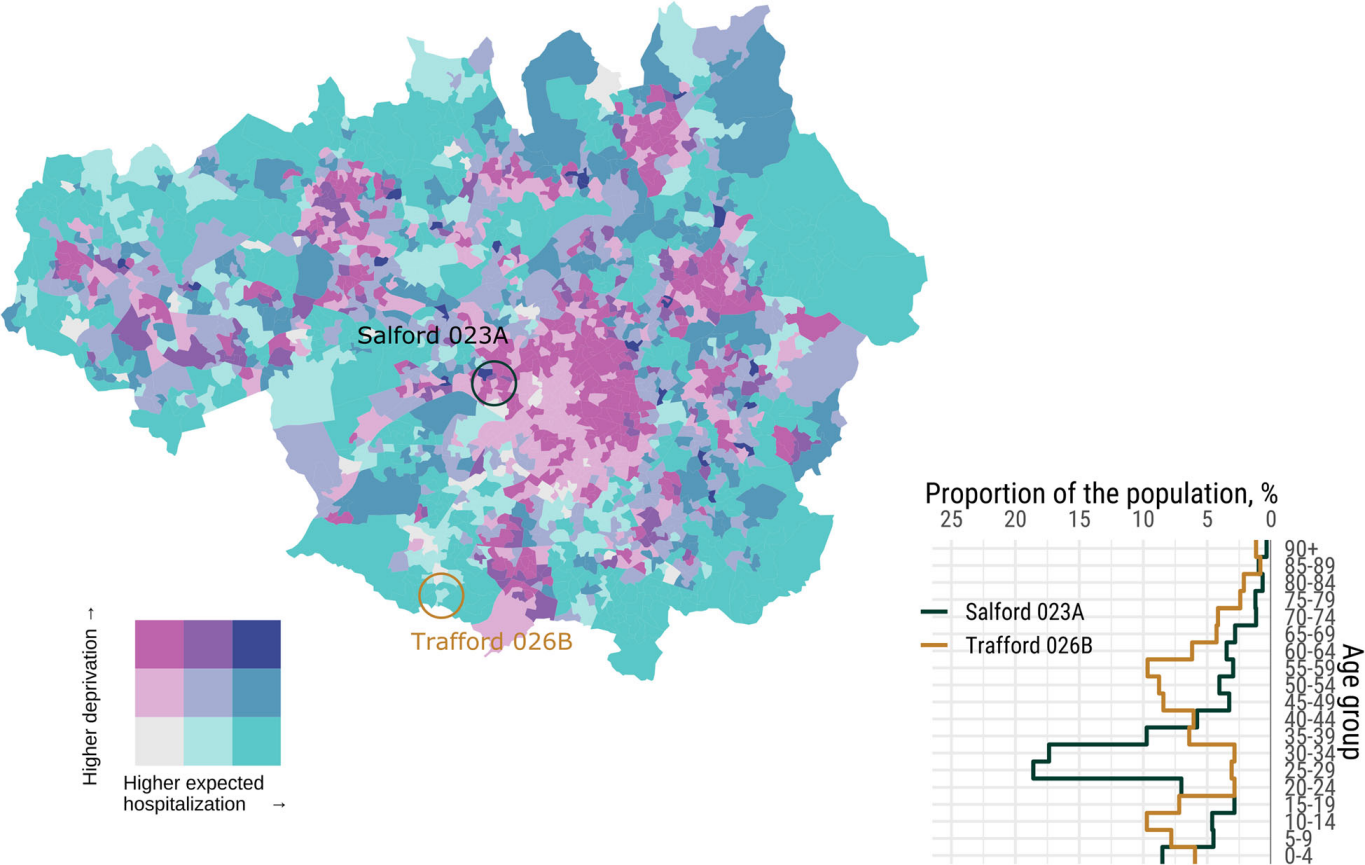
LSOA local differences in age-based hospitalization risk combined with social deprivation. Manchester

We also include bivariate maps to account for additional sociodemographic risks for COVID-19 in England and Wales. Figure 5 illustrates how age-based hospitalization risk measures interact with social deprivation and population density. These maps indicate that although population-based hospitalization risk tends to be lower in urban centres, higher levels of social deprivation and population density are conversely clustered around these same urban centres, which could counterbalance relatively low age-related risk levels. Figure 6 also visualizes telling nuances in the geographic risk in London, with a handful of areas having compounded risks of both high age-related hospitalization risks in addition to high levels of social deprivation. Figure 7 includes the proportion of ethnic minorities as the secondary axis, highlighting potentially higher health care pressures in different regions due to the disproportionate impact of COVID-19 on ethnic minorities [7]. Including such measures is important, as already evidenced by the limited data released on COVID-19 deaths at granular geographic levels. For example, the Middle Layer Super Output Area (MSOA) region of Newham 002 in London has a relatively low aged-based hospitalization risk yet it is one of the regions with the highest levels of social deprivation in England and had the highest proportion of deaths in the period between early March and mid-April [8]. Similarly, as COVID-19 spread to other areas such as Greater Manchester, ten COVID-19 related deaths were already reported in the MSOA region of Salford 002 in Greater Manchester over the same period, whereas none were recorded in, for example, Trafford 027 in Greater Manchester, even though the latter is characterized by a slightly older population (Fig. 8). Salford 002 is amongst the most socially deprived areas in the region, whereas Trafford 027 is prosperous.

## Discussion

Our results illustrate spatial differences in both expected hospitalization rates as well as the ability to cope with increased hospital demand given similar infection rates across regions, due to variation by key socioeconomic and demographic factors. Although local variations in hospitalization rates are scale invariant to the assumed overall infection rate, the actual spread of the infection depends on government policy, public adherence to policies and the unfolding of the initial virus and potential further peaks as lockdown eases. The companion online dashboard to this study affords flexibility to adjust changes in hospital capacity and overall infection rates as the pandemic continues to unfold.

It is likewise crucial to note that in response to the crisis and government’s realization of serious hospital bed shortages at the start of the pandemic, several interrelated processes in fact lowered the normal rate of hospitalization in England and Wales. Firstly, hospitals rapidly responded to the potential surge in demand and increased their own critical care bed capacity from normal pre-COVID-19 circumstances. This meant that the large numbers anticipated at the temporary ‘Nightingale’ hospitals did not materialize. Secondly, one-fifth of the £5 billion budget provided to the NHS to cope with COVID-19 was spent on increasing the rate of hospital discharge, with NHS England setting out the ambition to free up 30,000 of the 98,000 hospital beds—one-third of beds—in England [21]. This relates to the third reason, which is that NHS cancelled many routine elective operations resulting in a sudden drop in the demand for hospital beds. In fact, the NHSE guidance stated that 12,000 to 15,000 beds would be cleared by suspending elective procedures for 3 months [21]. Finally, there was the unexpected reaction that the public overwhelmingly stopped going to the hospital, even for serious conditions. Public Health England in fact showed a drop of around a third in visits to NHS emergency departments, starting as early as March 8, 2020, just 3 days after the first confirmed COVID-19 death on March 5 [22]. These developments coalesced to keep hospital demand manageable in the short-term, but especially as lockdown measures are eased and forms of normal life are resumed which will have the reverse effect on hospitalizations, it will become even more crucial to understand geographical differences in potential COVID-19-related health pressures.

## Conclusions

As countries across the globe exit strict lockdown and enter the ‘new normal’ of co-existence with COVID-19, monitoring new infection hotspots will be crucial. Our geospatial estimates illustrate the importance of considering demographic and socioeconomic factors in anticipating local spikes in health care demand related to the COVID-19 pandemic. The majority of existing models focus on aggregated national levels, which obscure regional differences [19]. Using the pre-crisis availability of hospital and critical care beds as a starting point and calibrating this to local demographic population composition and socioeconomic deprivation, we identify potential health care pressure points in England and Wales where expected hospitalization rates are disproportionately high and the per capita availability of hospital beds is relatively low. These spatial variations in underlying risk are key to inform disease monitoring in the coming months [11]. The early outbreak of COVID-19 in the UK was concentrated in densely populated urban areas with larger groups of ethnic minorities such as London. As of early May, there were higher levels of hospitalization due to COVID-19 in the north-west of England, particularly in Manchester and Liverpool. These areas have the highest levels of risk, including older populations, and higher rates of social deprivation and related comorbidities such as obesity and population density. In fact, as of early May, the region of Salford in Greater Manchester had one of the highest death rates in the country. Within this Borough are some of the most deprived wards in Greater Manchester, having the highest levels of unemployment and lowest life expectancy. As this pandemic continues to unfold across the world, we urgently need to consider how emerging sociodemographic risks such as social deprivation, race/ethnicity and population density structure spatial differences in COVID-19 severity and health care demand. In addition to health care preparedness, areas of high risk can be targeted for more aggressive testing and tracing to prevent transmission. Our online dashboard (https://covid19.demographicscience.ox.ac.uk/demrisk) provides a novel tool to integrate new knowledge of population risks in an agile way to assist real-time planning and prevention.

## Supplementary information

**Additional file 1: Fig. S1.** County baseline hospital bed capacity (per 1,000) for general care (A) and critical care (B). England & Wales

**Additional file 2: Fig. S2.** CCG baseline hospital bed capacity (per 1,000) for general care (A) and critical care (B). England

**Additional file 3: Fig. S3.** CCG expected hospitalization (per 1,000) for general care (A) and critical care (B) in case of a 10% overall infection. England

**Additional file 4: Fig. S4.** CCG excess need for hospital beds relative to baseline capacity (per 1,000) for general care (A) and critical care (B) in case of a 10% overall Infection. England

**Additional file 5: Fig. S5.** LSOA local differences in age-based hospitalization and local hospital capacity for general care (A) and critical care (B) in case of a 10% overall infection. Wales

## Data Availability

All data and code are available on our GitHub repository (https://zenodo.org/record/3723556). Our interactive dashboard that accompanies this article will allow policymakers to evaluate different thresholds of infection spreads, hospitalization rates and hospital bed capacity at the local level and is available at: https://covid19.demographicscience.ox.ac.uk/demrisk

## Abbreviations

CC: Ceremonial County
CCG: Clinical Commissioning Group
COVID-19: Coronavirus Disease 2019
LSOA: Lower Layer Super Output Area
MSOA: Middle Layer Super Output Area
NHS: National Health Service
NHSE: National Health Service England
ONS: Office of National Statistics
SDCS: Strategic Data Collection Service
