# Supplementary material for: Forecasting spatial, socioeconomic and demographic variation in COVID-19 health care demand in England and Wales
Source: BMC Med. 2020 Jun 29;18:203. doi: 10.1186/s12916-020-01646-2 (PMC7321716; doi:10.1186/s12916-020-01646-2)

**Supplementary Information 1**

**Figure S1. County baseline hospital bed capacity (per 1,000) for general care (A) and critical care (B). England & Wales**


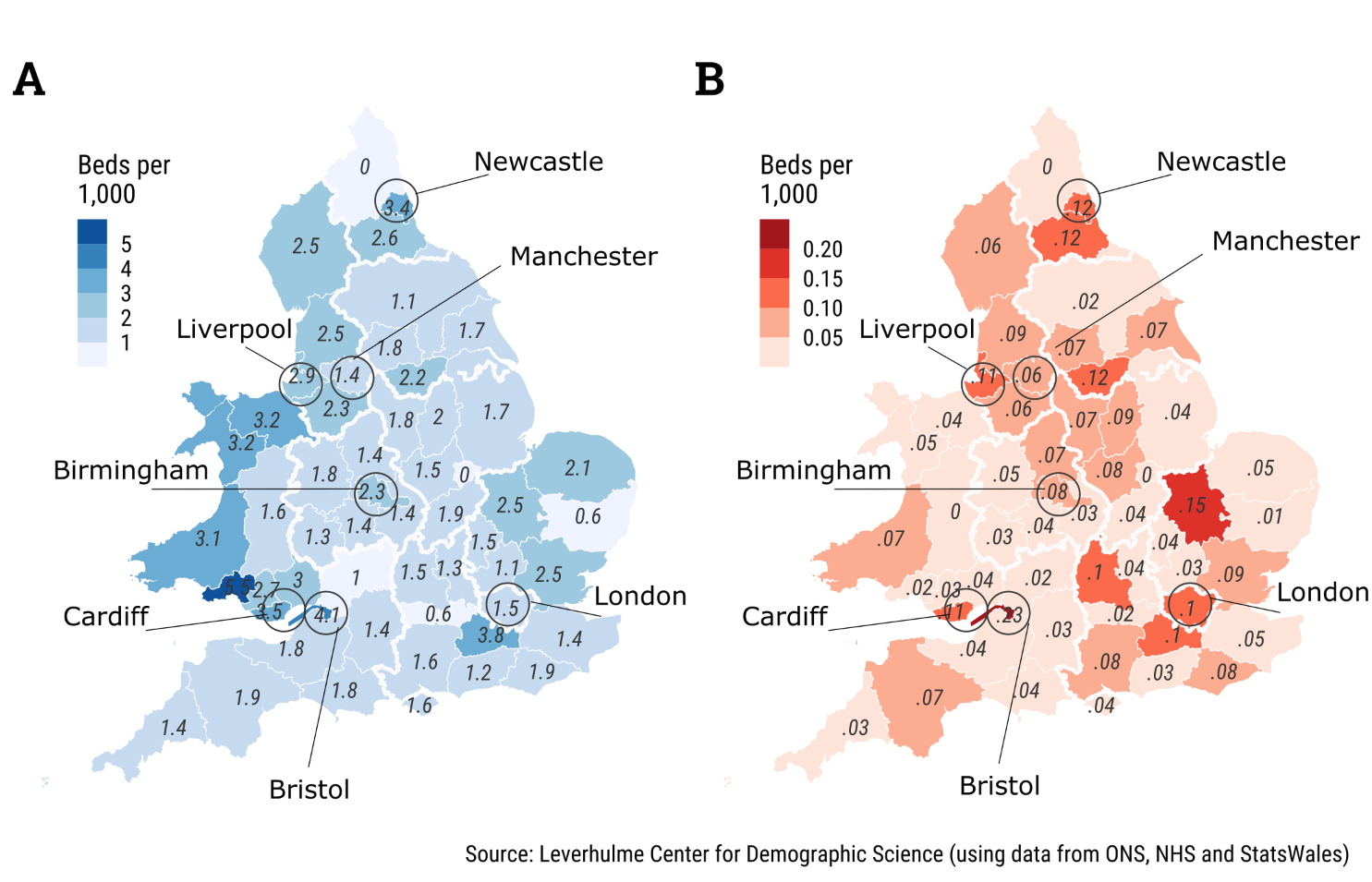
**Figure S2. CCG baseline hospital bed capacity (per 1,000) for general care (A) and critical care (B). England**


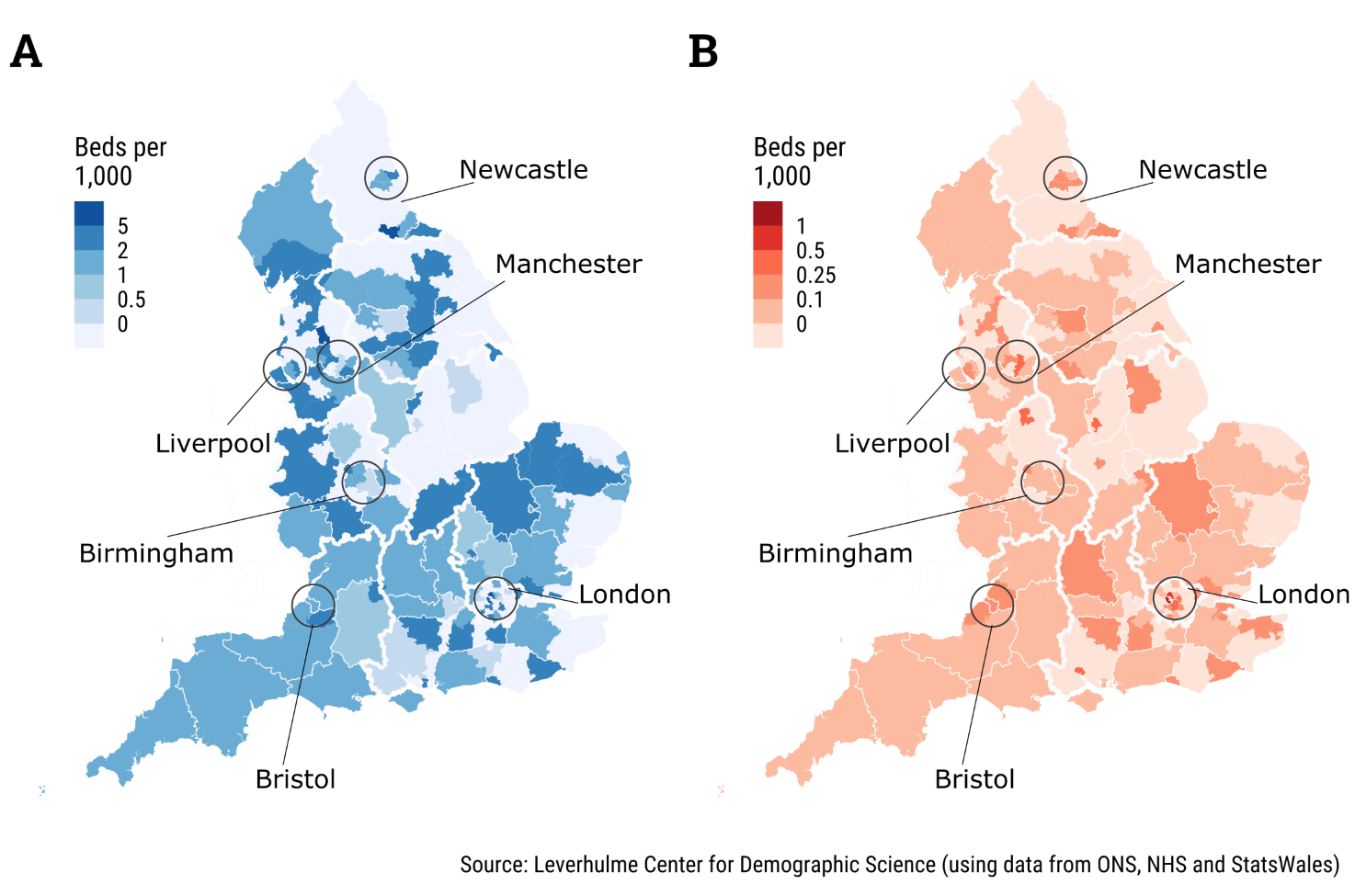


**Figure S3. CCG expected hospitalization (per 1,000) for general care (A) and critical care (B) in case of a 10% overall infection. England**


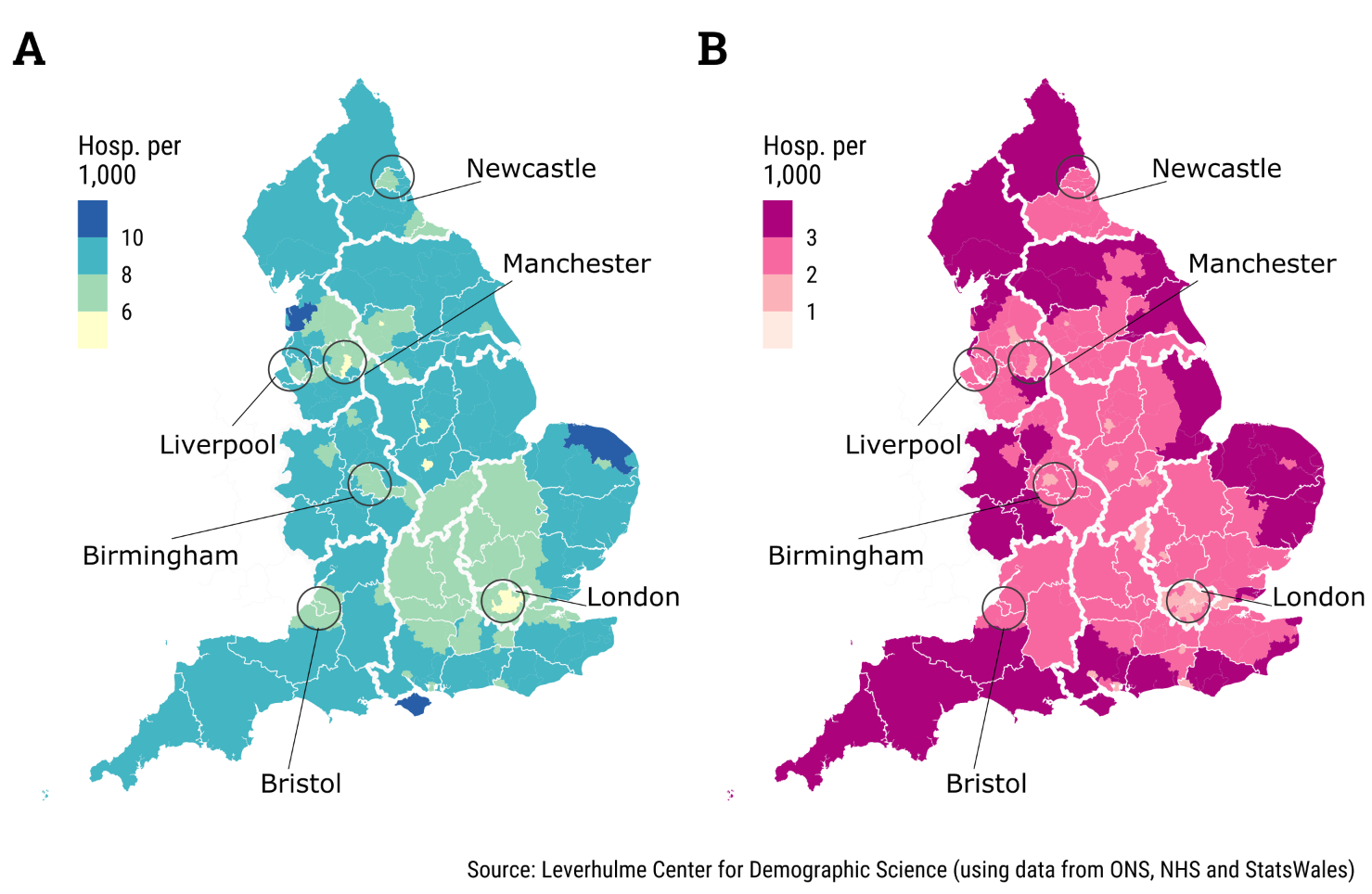


**Figure S4. CCG excess need for hospital beds relative to baseline capacity (per 1,000) for general care (A) and critical care (B) in case of a 10% overall Infection. England**


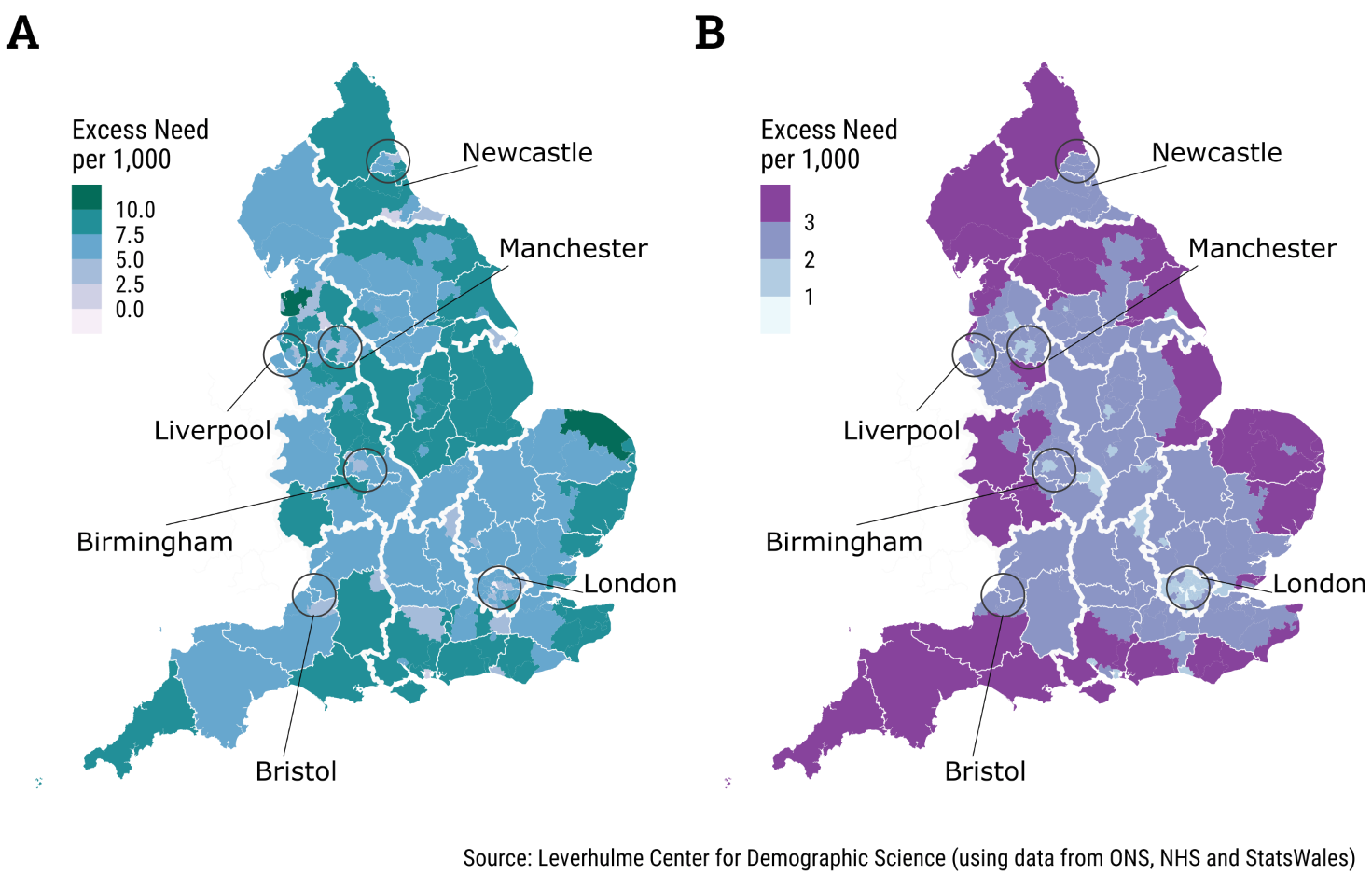


**Figure S5. CCG Excess Need for Hospital Beds Relative to Baseline Capacity (per 1,000) in Case of a 10% Nationwide Infection for General Hospitalization (A) and Critical Care (B). England**


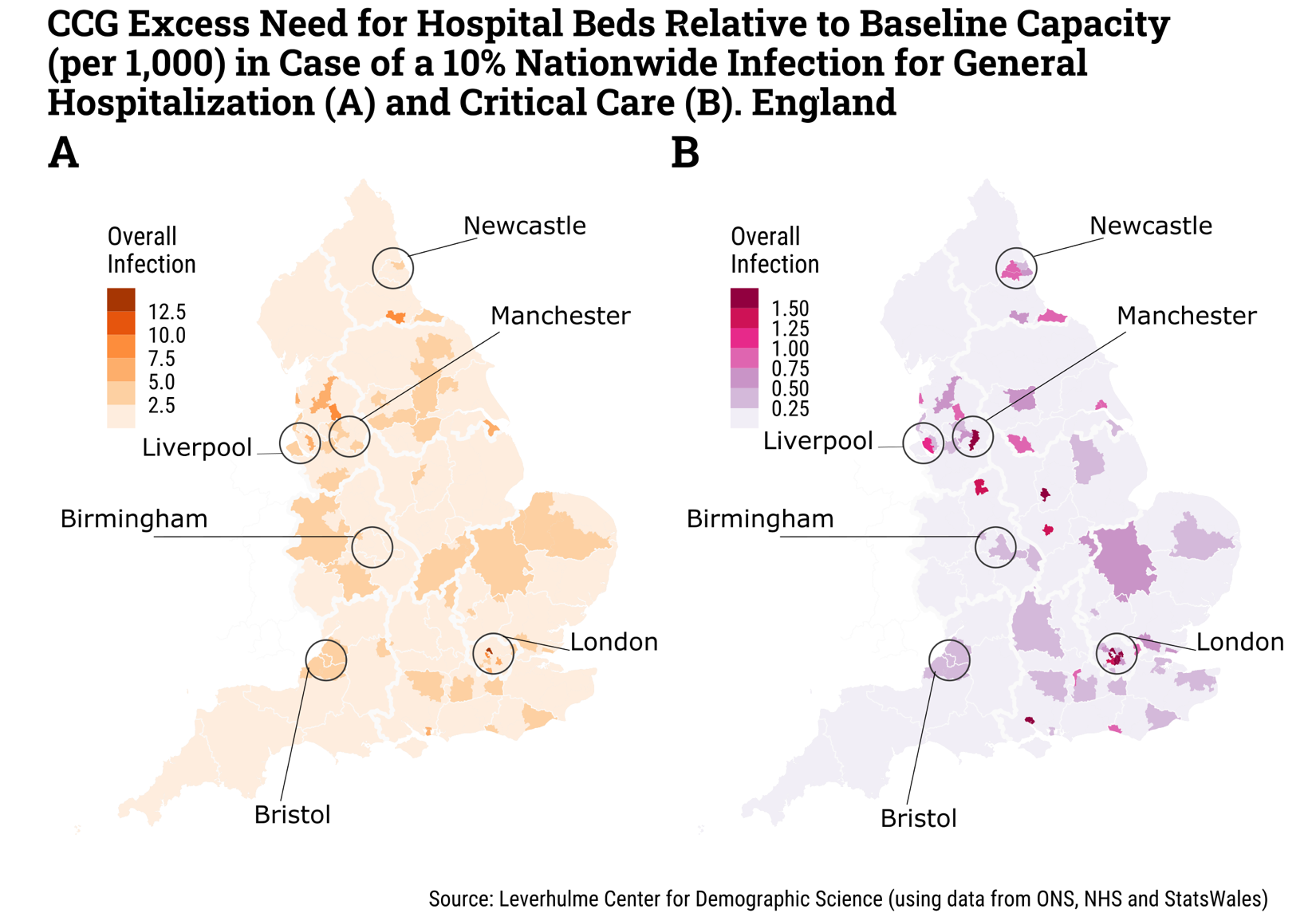


**Figure S6. LSOA local differences in age-based hospitalization and local hospital capacity for general care (A) and critical care (B) in case of a 10% overall infection. Wales**


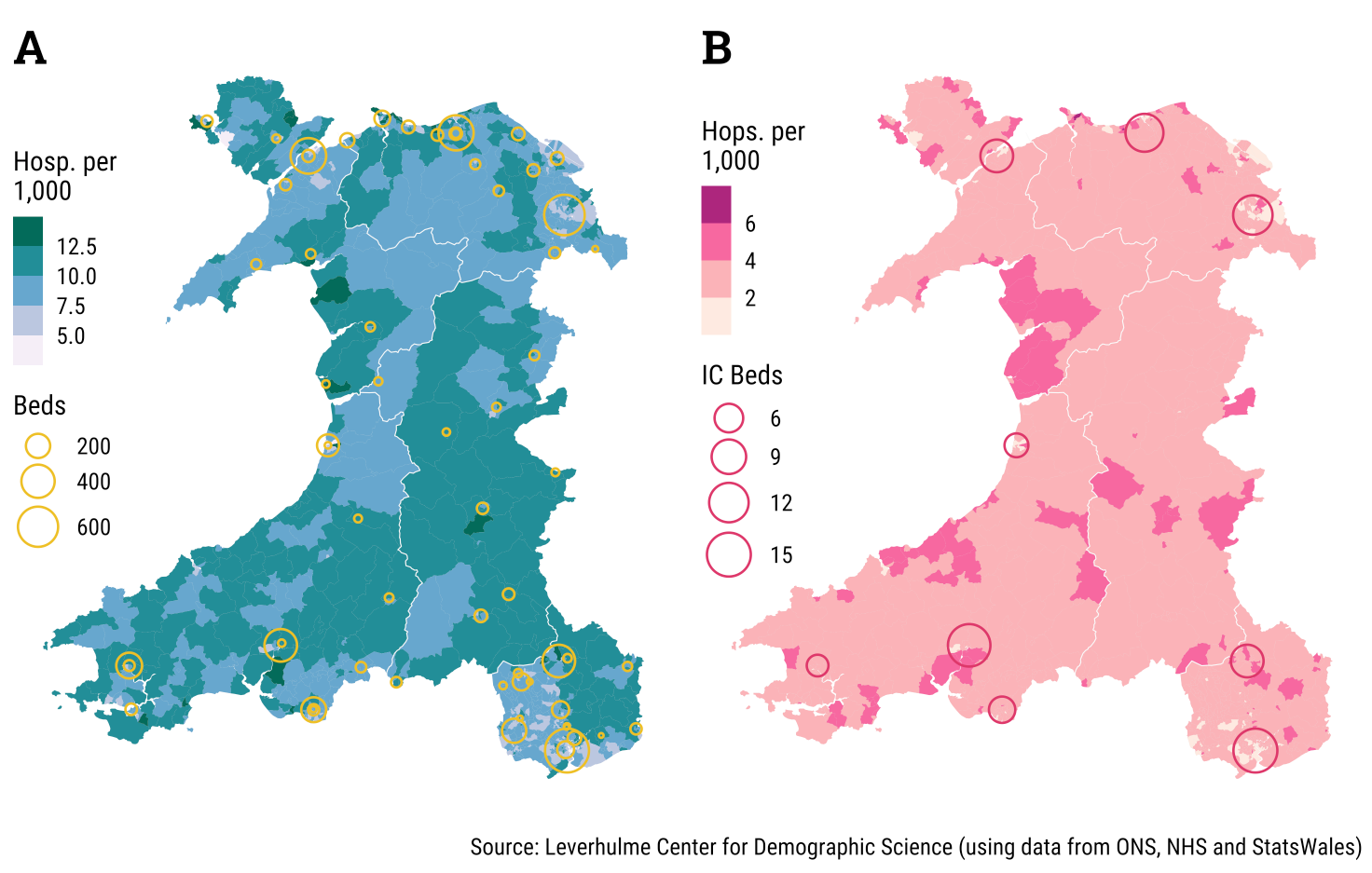

Supplement: Supplementary file 1 — Additional file 1: Fig. S1. County baseline hospital bed capacity (per 1,000) for general care (A) and critical care (B). England & Wales [file 12916_2020_1646_MOESM1_ESM.docx]
