# Supplementary figures and images for: Forecasting spatial, socioeconomic and demographic variation in COVID-19 health care demand in England and Wales
Source: BMC Med. 2020 Jun 29;18:203. doi: 10.1186/s12916-020-01646-2 (PMC7321716; doi:10.1186/s12916-020-01646-2)

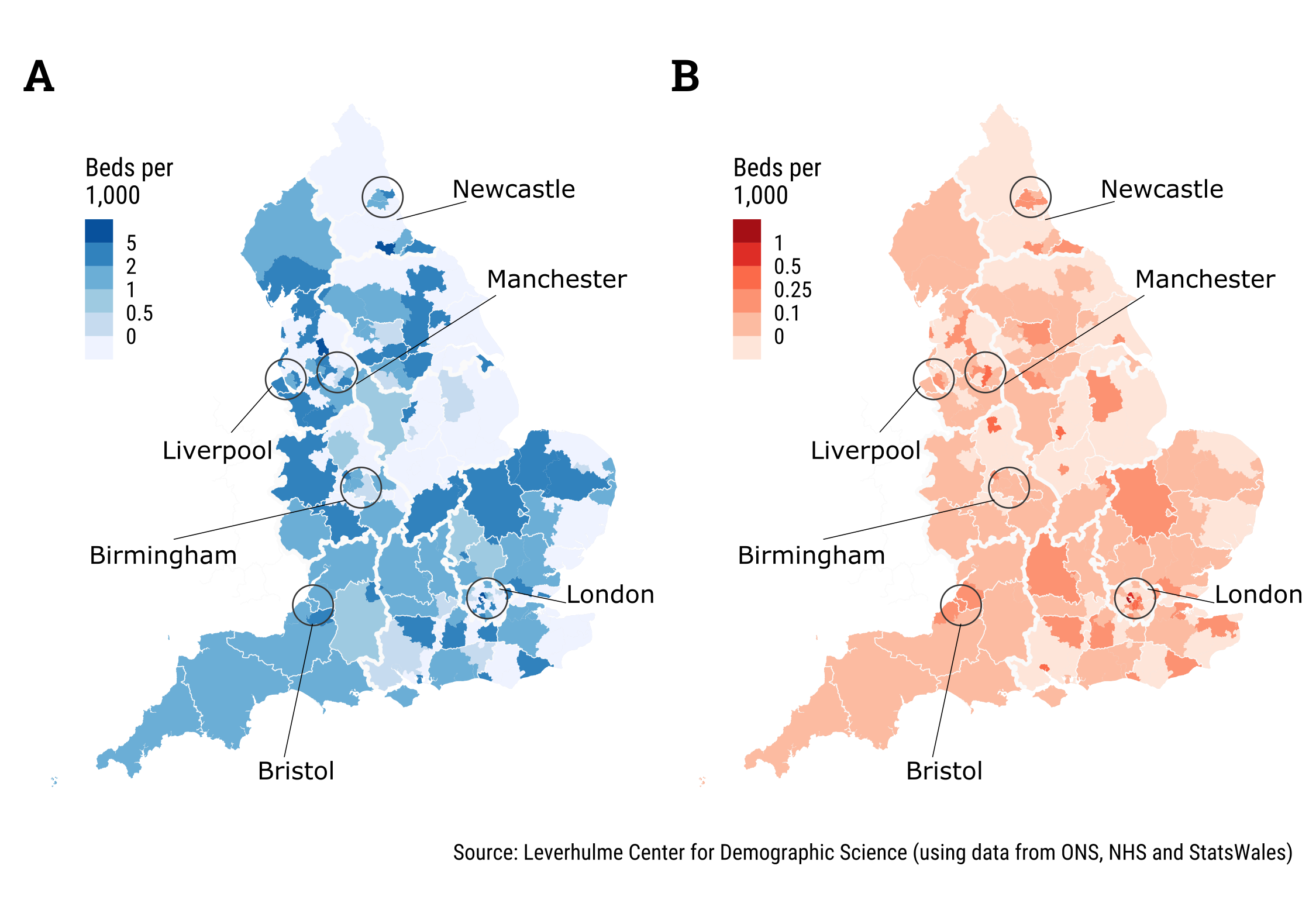

Supplement: Supplementary file 2 — Additional file 2: Fig. S2. CCG baseline hospital bed capacity (per 1,000) for general care (A) and critical care (B). England [file 12916_2020_1646_MOESM2_ESM.png]

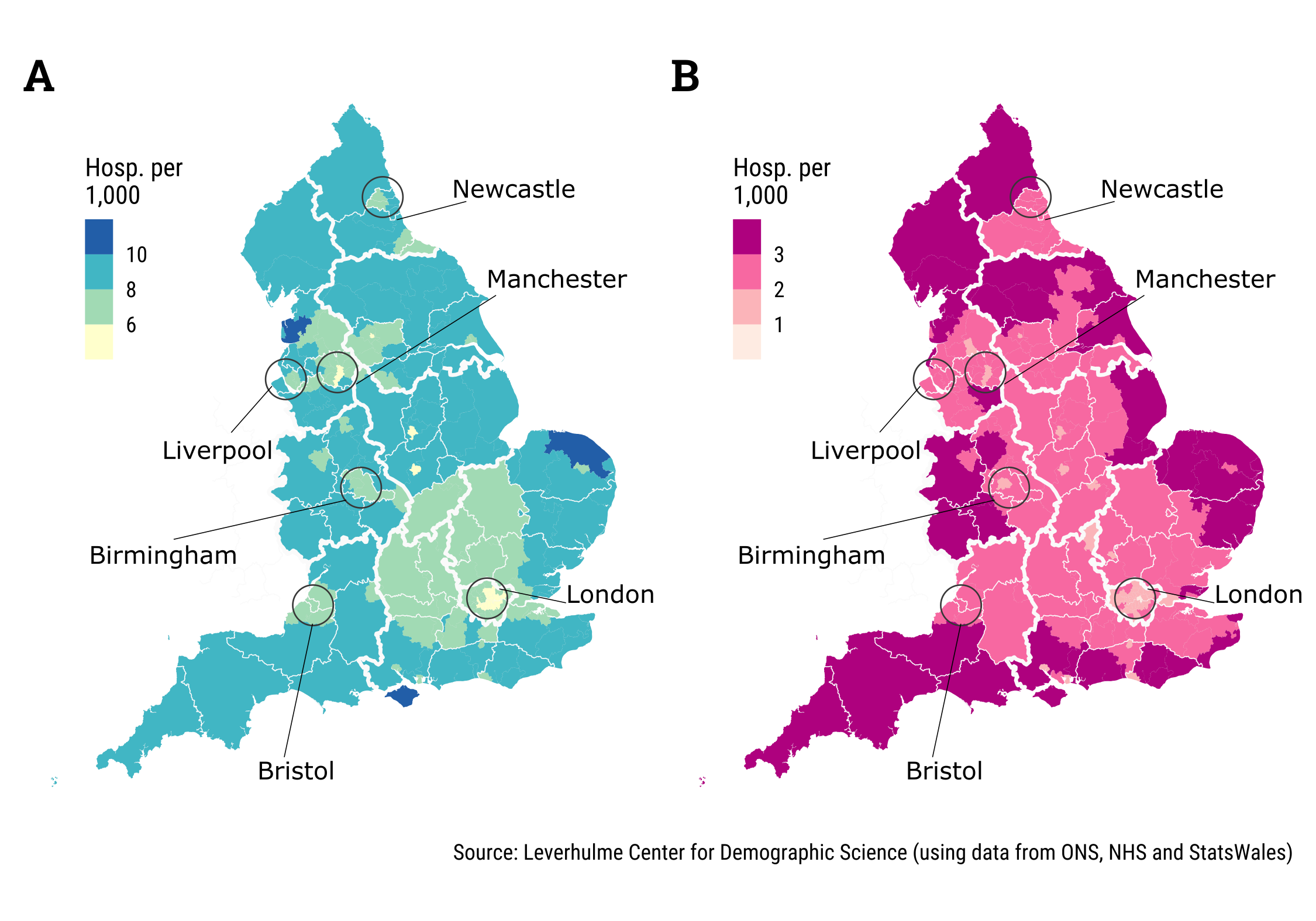

Supplement: Supplementary file 3 — Additional file 3: Fig. S3. CCG expected hospitalization (per 1,000) for general care (A) and critical care (B) in case of a 10% overall infection. England [file 12916_2020_1646_MOESM3_ESM.png]

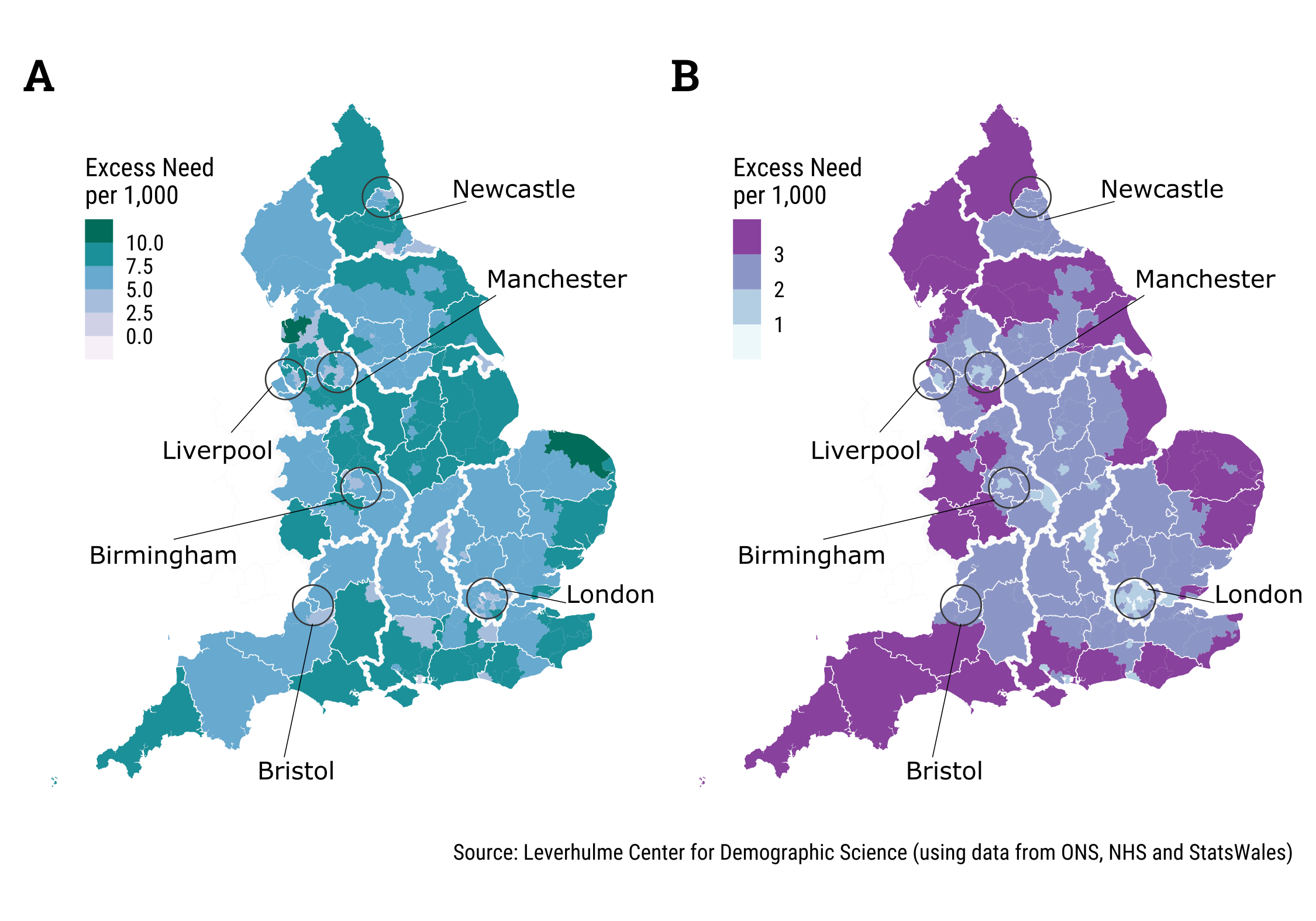

Supplement: Supplementary file 4 — Additional file 4: Fig. S4. CCG excess need for hospital beds relative to baseline capacity (per 1,000) for general care (A) and critical care (B) in case of a 10% overall Infection. England [file 12916_2020_1646_MOESM4_ESM.png]

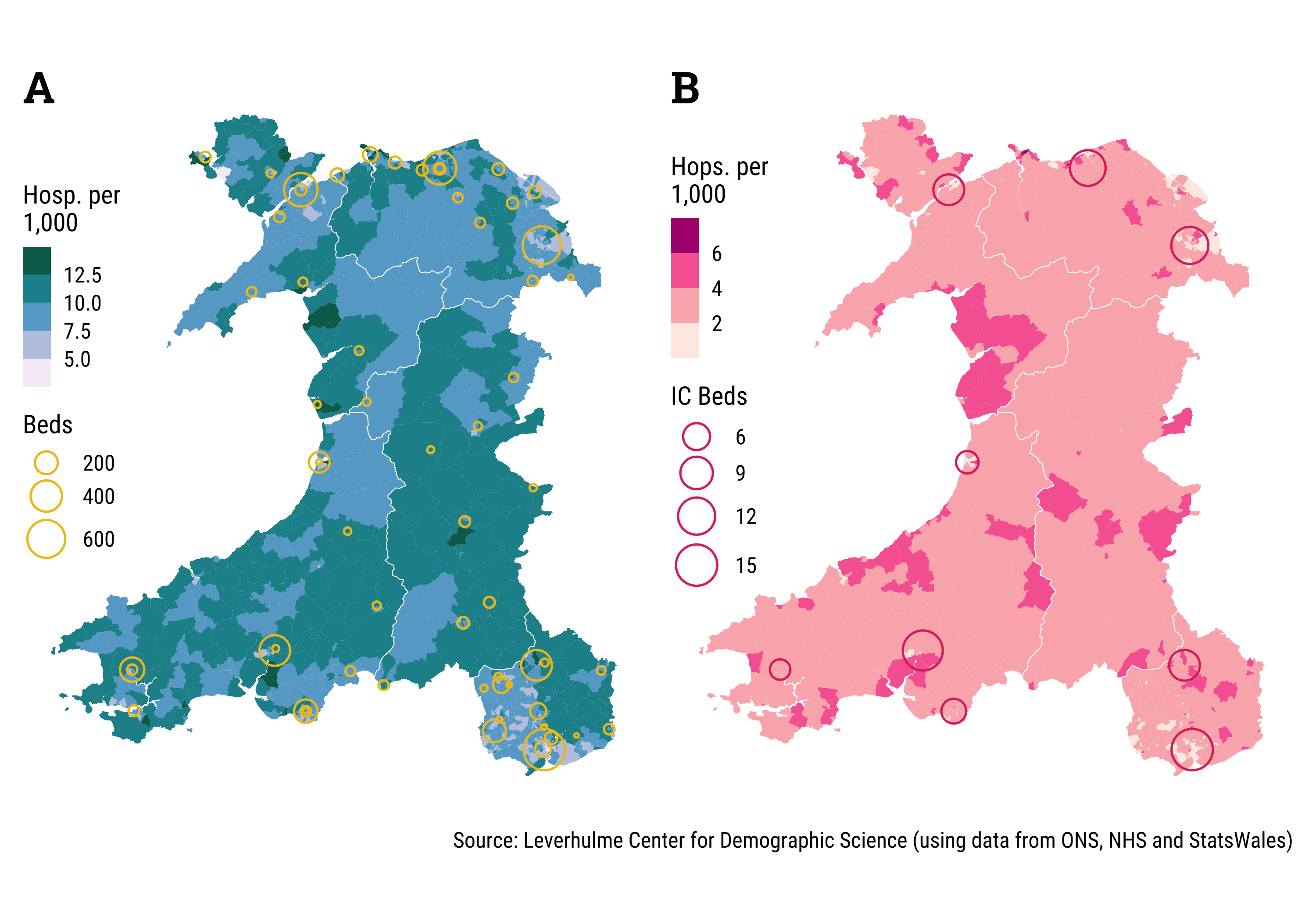

Supplement: Supplementary file 5 — Additional file 5: Fig. S5. LSOA local differences in age-based hospitalization and local hospital capacity for general care (A) and critical care (B) in case of a 10% overall infection. Wales [file 12916_2020_1646_MOESM5_ESM.png]
